# The effect of pamidronate delivery in bisphosphonate-naïve patients on neutrophil chemotaxis and oxidative burst

**DOI:** 10.1038/s41598-020-75272-6

**Published:** 2020-10-27

**Authors:** Jeffrey W. Chadwick, Howard C. Tenenbaum, Chun-Xiang Sun, Robert E. Wood, Michael Glogauer

**Affiliations:** 1 Department of Dental Oncology and Maxillofacial Prosthetics, Princess Margaret Cancer Centre, University Health Network, 610 University Avenue, Toronto, ON M5G 2M9 Canada; 2 Faculty of Dentistry, University of Toronto, 124 Edward Street, Toronto, ON M5G1G6 Canada; 3 Department of Dentistry, Centre for Advanced Dental Research and Care, Mount Sinai Hospital, 600 University Avenue, Toronto, ON M5G 1X5 Canada

**Keywords:** Innate immunity, Neutrophils, Oral manifestations

## Abstract

The pathogenesis of medication-related osteonecrosis of the jaw (MRONJ), a morbid condition associated with bisphosphonate administration, has not been fully elucidated. Recent research utilizing a murine model has revealed that the neutrophil becomes dysfunctional following exposure to bisphosphonates. Accordingly, the impairment of neutrophil function could play an important role in the pathogenesis of MRONJ via an infectious mechanism mediated by the suppression of the innate immune system. Currently, the existing human data are insufficient to substantiate this theory. To investigate, we isolated neutrophils from blood and oral rinse samples from bisphosphonate-naïve patients who were recently diagnosed with multiple myeloma both prior to and one month following their initial infusion of pamidronate, an intravenous bisphosphonate agent. Stimulated blood and oral neutrophil superoxide production and chemotactic capabilities were found to be impaired relative to baseline values. These results suggest that impaired neutrophil function may partially contribute to the aetiology underlying the pathophysiological processes linked to the development of MRONJ. Further, as the functional status of circulating neutrophils was reflected in the oral cavity where sampling can be accomplished in a non-invasive fashion, it is conceivable that neutrophil function could serve as a potential biomarker for MRONJ prognostication.

## Introduction

Bone is an organ that is frequently affected by the metastatic spread of several cancers^1^. Multiple myeloma, an incurable malignant plasma cell dyscrasia, is associated with bone metastases in approximately 90% of those patients diagnosed with this condition^2–4^. The pathogenesis of multiple myeloma is characterized by increased osteoclastic activity and osteoblast dysfunction which lead to localized bone resorption and skeletal-related events (SREs)^2^. SREs are debilitating comorbid conditions which include the development of bone pain, spinal cord compression and pathologic fractures^5^. The prescription of intravenous (IV) nitrogen-containing bisphosphonate (n-BP) medications in the setting of multiple myeloma is directed at the prevention of SREs^6^. The mechanism by which BPs exert their anti-metastatic effects extend beyond their capacity to attenuate osteoclastic differentiation, function and survival. In the management of multiple myeloma, IV n-BPs are capable of inhibiting neo-angiogenesis, dampening tumour cell adhesion and attenuating the production of local growth factors to reduce the establishment of metastatic foci of disease^7^.

While IV n-BPs such as pamidronate (PA) and zoledronic acid (ZA) are effective in preventing SREs in the setting of osseous metastases, these medications are associated with a number of adverse effects including acute-phase responses, ocular toxicity, nephrotoxicity, atrial fibrillation and cerebrovascular accidents^8^. One of the most significant conditions affecting patient quality of life (QoL), however, is medication-related osteonecrosis of the jaw (MRONJ)^9^. MRONJ, formerly termed bisphosphonate-related osteonecrosis of the jaw (BRONJ), is diagnosed in patients with a history of treatment with anti-resorptive or anti-angiogenic agents and bone which is either frankly exposed or that can be probed through cutaneous or intra-oral fistulae that has been present for a period of greater than eight weeks. Further, these patients must have not been treated with head and neck radiation therapy and be free from evidence of metastatic lesions involving the maxillae and mandible^10^. Clinical features of MRONJ are variable and include bone exposure, pain, swelling, intraoral, oroantral or cutaneous fistulae, and pathologic fractures of the mandible^11^*.* Despite the initial reports of MRONJ nearly two decades ago, the incidence of MRONJ secondary to IV n-BP administration in the setting of a malignant diagnosis is as high as 15% and evidence-based guidelines for management remain unavailable^12–14^. While the etiopathogenesis of MRONJ secondary to n-BP administration is uncertain, the underlying mechanisms suggested to account for the physical manifestations of this condition include alterations in osteoclastic function, soft and hard tissue toxicity and anti-angiogenic effects^15^.

Histopathologic examination of specimens collected from patients with MRONJ secondary to BP administration have revealed the presence of several bacterial species^16,17^. This finding suggests an infectious aetiology which may manifest via an impairment of the immune system, permitting the colonization of sites of exposed bone through persistent soft tissue defects. Interestingly, previous murine work has demonstrated that BPs possess the ability to perturb neutrophils, or polymorphonuclear leukocytes (PMNs), as evidenced by impaired PMN chemotaxis and nicotinamide adenine dinucleotide phosphate (NADPH) oxidase activity^18^. PMNs represent a heterogenous population of cells capable of remarkable functional and phenotypic plasticity^19^. As an integral member of the innate immune system, PMNs are recruited from the circulation and migrate through tissues along chemoattractant gradients to clear invading microorganisms via phagocytosis, degranulation, neutrophil extracellular trap (NET) release and reactive oxygen species (ROS) production^20^. Regulation of PMN chemotaxis and the aforementioned antimicrobial effector functions is dependent on the prenylation of several small GTPases which act as mediators of intracellular signalling pathways^21^. The downstream inhibition of these same GTPases in osteoclasts secondary to n-BP exposure is responsible for the desired inhibition of osteoclastic function via dysregulation of cytoskeletal organization, loss of ruffled borders and attenuation of vesicular trafficking of critical lysosomal enzymes^22^. While the specific role of PMNs in the etiopathogenesis of MRONJ remains unclear, if n-BPs are capable of altering the prenylation of the aforementioned cellular targets within PMNs in a manner analogous to that which affects osteoclasts, IV n-BP treatment may lead to a functional impairment of the innate immune system through decreased PMN activity.

In the present study, we evaluate the effect of a single administration of an IV n-BP on human blood and salivary PMNs collected from BP-naïve patients diagnosed with multiple myeloma. This work represents the largest human study to assess PMN function in the context of IV n-BP administration. PMN function was assessed both before and one month following an initial PA infusion utilizing in vitro chemotaxis and ROS production assays. Blood and salivary PMN function were assessed in tandem to determine if systemic PMN impairment was reflected in peripheral tissues. A non-invasive method for testing PMN function in patients receiving n-BP therapy may be developed if oral and blood PMNs are affected similarly following treatment with PA.

## Results

### Study population

A total of 14 BP-naïve patients diagnosed with multiple myeloma were recruited during the one-year study period. Three patients were deemed ineligible due to sample collection failure, delayed refusal of PA therapy and development of a severe allergic reaction to trimethoprim/sulfamethoxazole (TMP/SMX) at a time proximal to post-PA sample collection. Eleven patients (eight males and three females) with a mean age of 54.8 ± 13.0 years met the inclusion criteria and were included in the analysis (Table 1). At the time of all sample collections, patients were free from neutropenia as well as evidence of either oral and systemic infection. All patients received an initial IV n-BP infusion (90 mg) of PA between sample collections.

**Table 1:** Bisphosphonate-naïve study patient information.

Age	Sex	Medical history	Pending multiple myeloma therapy
76	M	Hypertension	Revlimid, Dexamethasone
35	M	None	None
48	F	Asthma	CyBorD
40	M	Ankylosing Spondylitis	CyBorD CyBorD
54	M	Dyslipidemia	CyBorD
55	F	Hypertension, Dyslipidemia, Diabetes	CyBorD
34	M	None	CyBorD
58	M	Arthritis	None
64	M	Atrial Fibrillation, Dyslipidemia	Bortezomib, Dexamethasone
77	M	Coronary Artery Disease	CyBorD
62	F	Sickle Trait	CyBorD

CyBorD: Cyclophosphamide, Bortezomib, Dexamethasone.

### Respiratory burst

**Figure 1:**
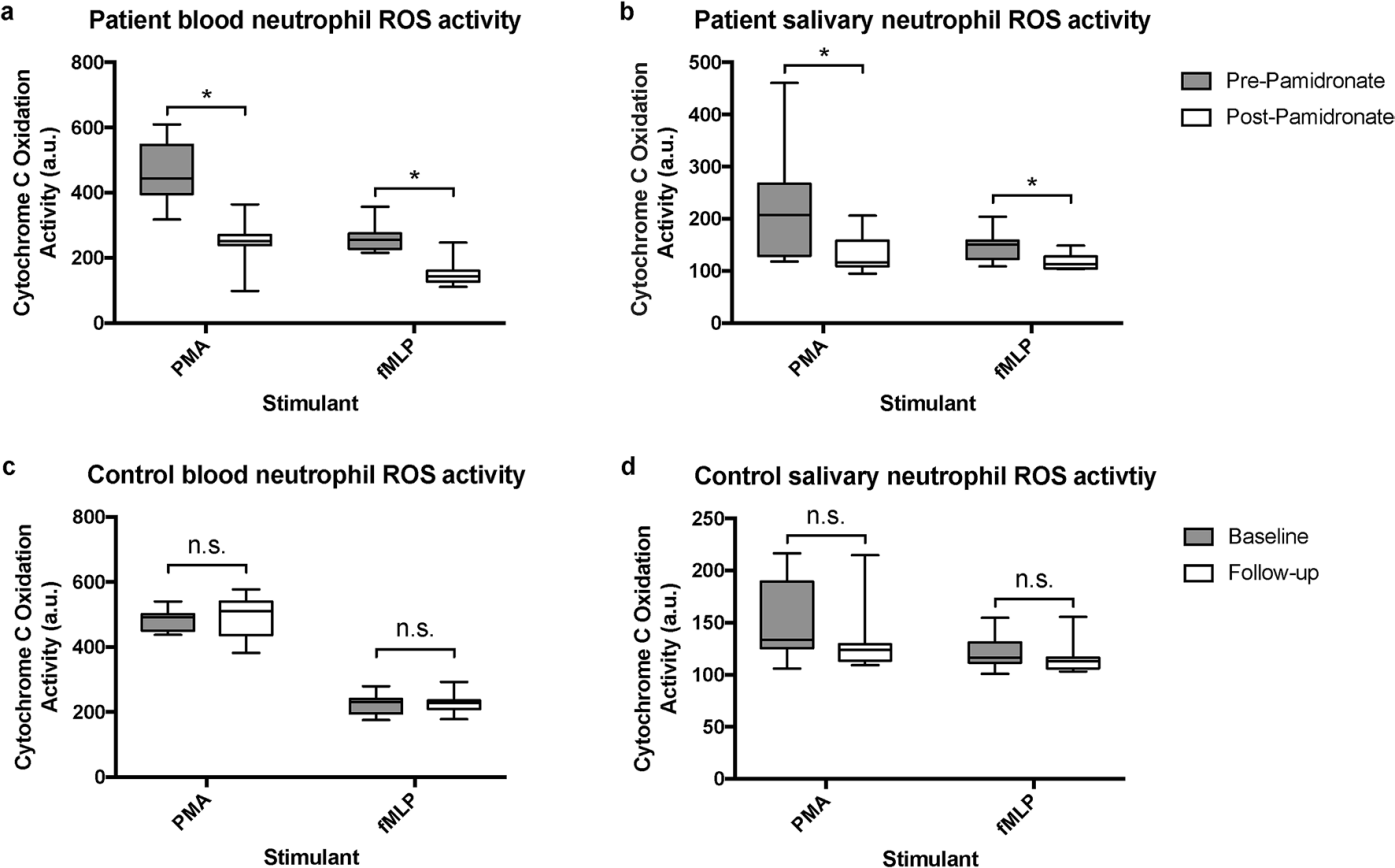
Blood and salivary neutrophil ROS activity. Boxplots of patient blood (**a**), patient salivary (**b**), control blood (**c**) and control salivary (**d**) PMN ROS activity as measured by cytochrome c oxidation following PMA and fMLP stimulation prior to and one month following an initial administration of IV PA for patients and at baseline and one-month follow-up time points for controls. Stimulation of 1 × 10^6^ recovered blood and salivary PMNs. Boxes represent interquartile range, line represents median, whiskers represent maxima and minima. *p ≤ 0.05; n.s.: Not statistically significant, *p* > 0.05; a.u.: Absorption units; IV PA: Intravenous pamidronate; PMA: Phorbol myristate acetate; fMLP: N-formyl-methionyl-leucyl-phenylalanine; ROS: Reactive oxygen species.

Patient exposure to single infusion of PA resulted in a significant reduction in PMN ROS production (Fig. 1a,b). Compared to pre-infusion production, a PA-induced decrease in ROS production of 41.2% and 19.5% by patient blood and oral PMNs, respectively, in response to fMLP stimulation was noted (*p* ≤ 0.05). A similar response to PMA stimulation was noted, resulting in a PA-induced reduction in ROS production of 45.5% and 41.4% for patient blood and oral PMNs, respectively (*p* ≤ 0.05). There were no statistically significant differences between external control samples regardless of the applied stimulant (*p* > 0.05) (Fig. 1c,d).

### Chemotaxis

**Figure 2:**
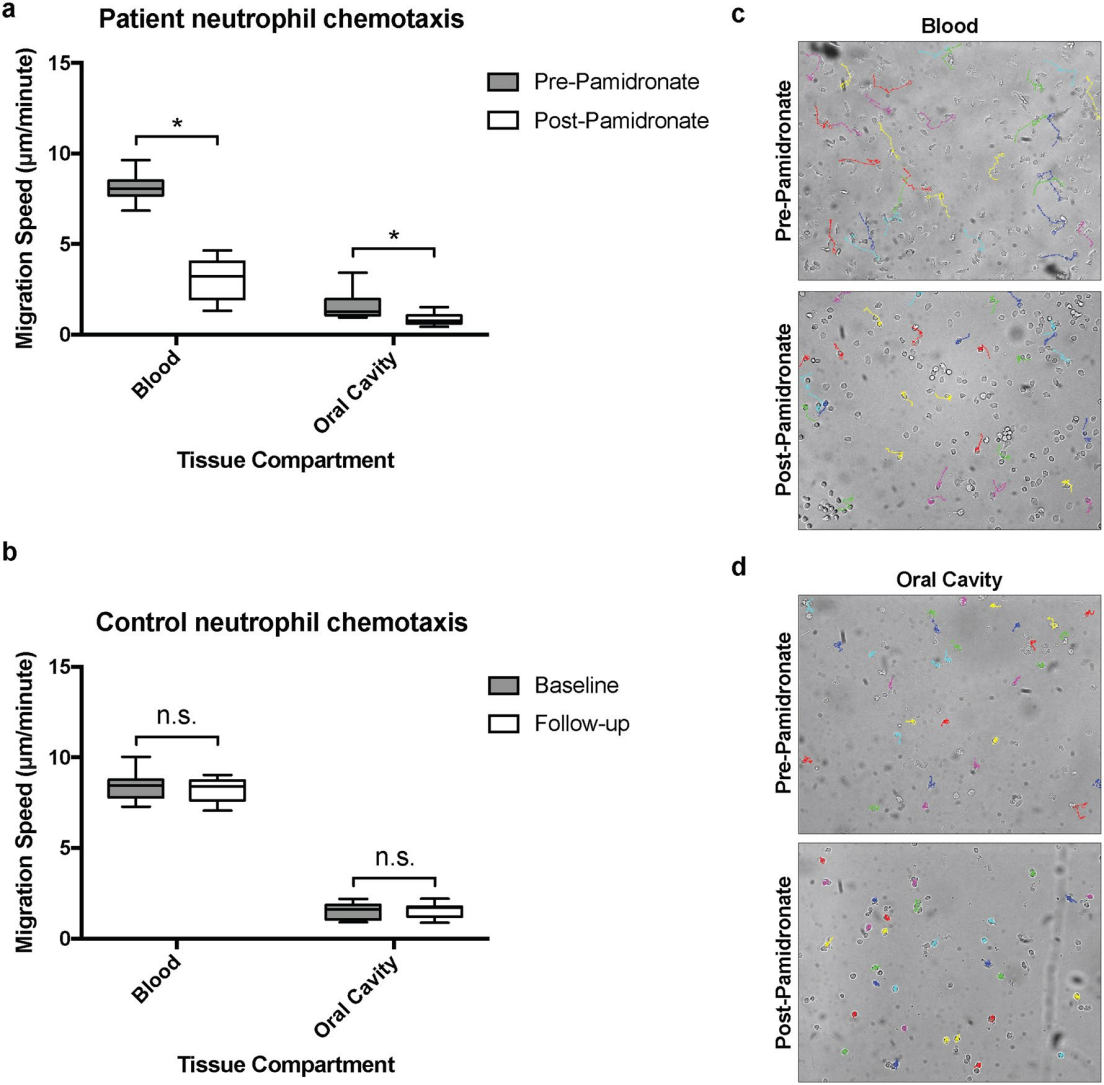
Blood and salivary neutrophil chemotactic activity. Boxplots of patient (**a**) and control (**b**) blood and salivary PMN chemotactic activity as measured within a Zigmond chamber following fMLP stimulation prior to and one month following an initial administration of IV PA for patients and at baseline and one-month follow-up time points for controls. Photomicrographs of patient blood (**c**) and salivary (**d**) PMN chemotaxis within a Zigmond chamber toward an fMLP stimulus (right side of photomicrograph) prior to and one month following an initial administration of IV PA (magnification 20X). Stimulation of 1 × 10^6^ recovered blood and salivary PMNs. Boxes represent interquartile range, line represents median, whiskers represent maxima and minima. *p ≤ 0.05; n.s.: Not statistically significant, *p* > 0.05; IV PA: Intravenous pamidronate; fMLP: N-formyl-methionyl-leucyl-phenylalanine; μm: Micrometre.

The migration of PMNs toward an fMLP stimulus was deficient in patients treated with PA. Reduction in chemotactic function was observed for both salivary and blood PMNs (Fig. 2a). Overall, there was a 61.4% and 49.1% reduction in blood and oral PMN chemotaxis, respectively (*p* ≤ 0.05). No statistically significant difference was noted in the control analysis (*p* > 0.05) (Fig. 2b).

## Discussion

This is the first controlled human study evaluating the effects of IV n-BP administration on PMN function. Results from this work demonstrate a marked impairment of both blood and oral PMN ROS production and chemotactic capabilities following an initial administration of PA. These features are noteworthy as they suggest an acute suppression of the innate immune system which may have deleterious consequences for patients requiring long-term prescription of IV n-BPs. This is also the initial report demonstrating the successful application of a non-invasive oral rinse for PMN isolation and functional testing in the setting of IV n-BP administration.

There is a growing body of literature characterizing the impact of BPs on both the innate and adaptive immune system. As a functional immune system is proposed to be a vital factor in the development of MRONJ, this has been an area of active investigation. Recent in vitro studies have demonstrated the reduction of macrophage viability and monocyte differentiation as well as increased matrix metalloproteinase expression in the setting of BP exposure utilizing a THP-1 human monocytic cell model^23,24^. Further, analyses of osseous specimens from patients diagnosed with MRONJ have also demonstrated a marked increase in both macrophage infiltration as well as polarization toward a destructive M1, or classically-activated, phenotype mediated by BP exposure which may impair bone tissue homeostasis^25^. The adaptive immune response is, unfortunately, not unscathed by the action of BP therapy as suppression of γδ T cells has been reported. This suppression occurs due to PMN uptake of n-BPs and subsequent production of serine proteases, arginine I and hydrogen peroxide as well as decreased production of TNFα and IL-8. This leads to diminished γδ T cell expansion and hampering of the cytolytic capabilities of these cells, which are paramount in the setting of malignant disease^26^.

Observations from our current study revealed that treatment with a single dose of an IV n-BP affects neutrophil motility and ROS production. IV n-BPs, such as PA, act along the mevalonate pathway, inhibiting the action of farnesylpyrophosphate synthase (FPPS) resulting in decreased downstream production of geranylgeraniol pyrophosphate (GGP) which is required for the prenylation of several GTPases within the osteoclast. Inhibiting GTPase prenylation results in the loss of several cellular functions including the modification of the cytoskeleton and binding to osseous surfaces culminating in the inhibition of osteoclastogenesis as well as the induction of apoptosis^22^. We hypothesize that these same mechanisms are likely to manifest within the neutrophil, as previous reports demonstrate a loss of PMN chemotactic and NADPH oxidase activity in the setting of GTPase prenylation inhibition^21^. In fact, previous work by our group has demonstrated that decreases in Rho GTPase activity are associated with impaired PMN migration^27,28^. While the current literature does not directly inculpate PMN dysfunction as a major constituent of MRONJ pathogenesis, recent data from our group using a murine model demonstrated that n-BP exposure hampers vital PMN functionality^18^. In these studies, we observed that both ZA and PA dampen PMN chemotaxis after in vitro and in vivo exposure while PMN ROS production is diminished in vitro. Further, in vitro ZA treatment resulted in a nearly 50% depression in PMN fMLP-mediated Rho small GTPase activity. Interestingly, the previously noted effects on murine PMN chemotaxis were found to persist at four weeks following n-BP exposure which is consistent with our current results^18^. Other human studies conducted by our group have corroborated these findings, demonstrating both decreased PMN ROS production and chemotactic capabilities in patients with an established diagnosis of MRONJ secondary to n-BP exposure and, in a limited number of patients without BRONJ, following n-BP exposure relative to healthy controls^29^**.** Curiously, others have demonstrated that in vitro exposure to n-BPs increase human PMN ROS production^30^. This effect was noted, however, after only 16 h of BP incubation which is in stark contrast to the four weeks of in vivo exposure in our study with their remaining findings regarding human PMN chemotaxis aligning with our results. Resultantly, it should follow that impaired PMN recruitment and reduced lysosomal functionality may facilitate delayed wound healing, maintenance of soft tissue defects and failure to control local infections. These functional deficits may lead to the establishment of MRONJ and, ultimately, support an infectious aetiology for this disease process^29^.

The generalizability of the results from these investigations are limited by a number of factors. First, during the recruitment period for this study, PA was the primary BP prescribed to treat those individuals with evidence of skeletal involvement. Although there have not been any well-controlled studies which demonstrate improved overall survival, ZA has since replaced PA in the context of the treatment of multiple myeloma due to its association with a decreased rate of SREs and reduced infusion time^31–33^. It would be prudent to ensure that the results discussed here are recapitulated in the setting of treatment with ZA which is likely as ZA is of the same BP class as PA. Second, although the institution from which patients were recruited treats a large volume of patients afflicted with multiple myeloma, recruiting individuals whom were bisphosphonate-naïve proved challenging for a myriad of reasons. The authors recognize that the study participants varied widely in age and several were exposed to cytotoxic agents and corticosteroids for the management of their underlying malignant condition. The aforementioned agents are capable of suppressing PMN function and production and immunosenescence has profound effects on the functioning of the innate and adaptive immune system. To control for these potential confounders, patients served as their own controls and all sample collections were performed immediately before subsequent cycles of chemotherapy with complete blood counts demonstrating normal absolute neutrophil counts^34–36^.

The results from this work raise a number of important questions which can be investigated in future studies. First, patients receiving IV n-BP therapy should be monitored for extended periods to determine if PMN impairment persists both during and following the termination of IV n-BP treatment. This information is integral as the risk of MRONJ is known to persist beyond the initial BP administration period due to the protracted half-lives of these compounds secondary to long-term osseous binding and continuous release during skeletal turnover^37^. This phenomenon would lend significant weight to the contribution of a dysfunctional immune response in MRONJ pathogenesis. Second, a practical application predicated on impaired PMN function as a biomarker which could be facilitated in a non-invasive manner as was demonstrated in this study is of immense interest. Although numerous studies have resolved associations between MRONJ and a variety of bone turnover markers, the current body of literature suggests that the available biomarkers can neither accurately diagnose or prognosticate MRONJ^38^*.* Our data, while intriguing, does not directly support the use of PMN functionality as a biomarker for MRONJ as we have only observed PMN dysfunction after the initial administration of PA. Moving forward, it will be critical to prospectively compare PMN function between those individuals who develop MRONJ and those who do not after receiving IV n-BP therapy to determine if there is a minimal decrease in baseline PMN function which places patients at significant risk of developing this morbid condition.

## Conclusions

The results from this study demonstrate that PMN chemotaxis and ROS production are impaired following the administration of PA in previously BP-naïve patients. These findings lend evidence to suggest a potentially infectious aetiology for MRONJ. Future work regarding the maintenance of PMN functional impairment is paramount to further elucidate the mechanisms which govern the pathogenesis of MRONJ. Further, the potential use of PMN function as a biomarker for MRONJ susceptibility is integral to risk stratification and clinical management of this patient population.

## Methods

### Study population

**Figure 3:**
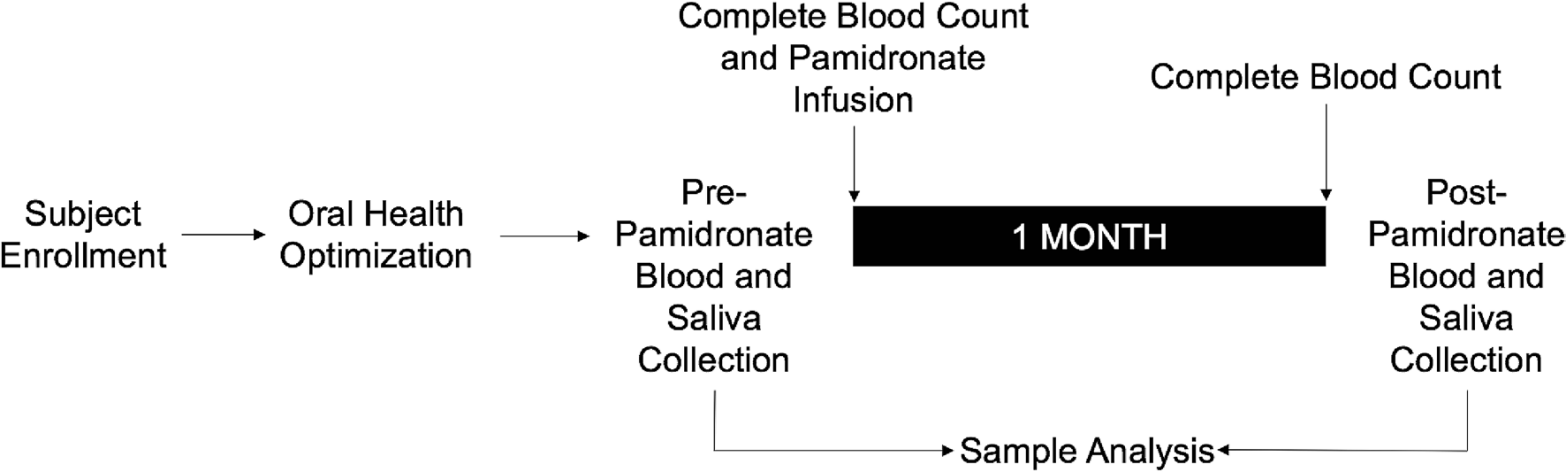
Study design. Following enrolment, oral health of study participants was optimized. Complete blood counts were performed before all sample collections. *Note*: Samples from a healthy volunteer were collected at both pre-pamidronate and post-pamidronate time points as an external control for chemotaxis and reactive oxygen species assays.

Patients satisfying the inclusion criteria of the study were consecutively enrolled for participation between July 2014 and July 2015 (Table 2). Following oral health optimization, blood and oral rinse specimens from each subject, as well as a single healthy 33-year-old male who served as an external control, were collected at each pre-defined interval (Fig. 3). Patients and the control were noted to be free from signs and symptoms of infection during the study period as well as at the time of sample collection. Patients were assigned a unique patient identifier to blind both lab personnel and data analysts. All samples were immediately processed to assess PMN function and analysed simultaneously with the external control. Ethical approval was obtained from the University Health Network (UHN) Research Ethics Board (10-0936CE). Informed consent was obtained from all study participants in both verbal and written formats. This study, including sample collection and sample processing, was conducted according to the guidelines set by the Declaration of Helsinki.

**Table 2:** Study inclusion and exclusion criteria.

Inclusion	Exclusion
18 years of age or older	Younger than 18 years of age
New diagnosis of multiple myeloma	Incapable of providing informed consent
No previous history of bisphosphonate administration	Previous bisphosphonate administration (oral or IV)
Starting intravenous pamidronate (90 mg/month)	Pregnant (any trimester)
Capable of providing informed consent	Life expectancy less than 1 month
	Uncontrolled diabetes mellitus (HgbA1C > 8.0%)
	Previous head and neck radiation therapy

HgbA1c: Glycosylated haemoglobin; IV: Intravenous; mg: Milligram.

### Blood sample preparation

Blood samples were collected by venepuncture from the median cubital vein into two 5 mL sodium citrate vacutainers (Becton Dickinson). PMNs were isolated using 1-Step Polymorphs (Accurate) according to the manufacturer’s instructions. Residual erythrocytes were lysed with 1 mL of distilled water and recovered PMNs were washed in Hank’s balanced salt solution without calcium and magnesium (HBSS^-/-^, Gibco) and centrifuged at 1500 RCF for five minutes. The supernatant was discarded and the PMN pellet was resuspended in 1 mL phosphate buffered saline without calcium and magnesium (PBS^-/-^, Sigma-Aldrich). Recovered blood PMNs were quantified using a Z2 Cell and Particle Counter (Beckman Coulter) and demonstrated greater than 95% cell viability and 98% purity as assessed by a trypan blue exclusion test and Kwik-Diff staining (Thermo Fisher Scientific), respectively.

### Oral rinse preparation

Patients were instructed to perform five 30 s oral rinses separated by one minute with 3 mL of sterile 0.9% normal saline (Baxter). The oral rinse samples were deposited into a 50 mL conical tube (Becton Dickinson) and immediately subjected to sequential filtration utilizing a sterile 40 μm nylon mesh sterile cell strainer (Fisher Scientific) followed by 20 μm and 11 μm nylon net filters (Millipore). The filtrate was centrifuged for 10 min at 1500 RCF and the cell pellet was resuspended in 1 mL of sterile PBS^-/-^. Recovered salivary PMNs were quantified using a Z2 Cell and Particle Counter (Beckman Coulter) and demonstrated 95% cell viability and 98% purity as assessed by a trypan blue exclusion test and Kwik-Diff staining (Thermo Fisher Scientific), respectively.

### PMN superoxide production

A suspension of 1 × 10^6^ PMNs in 100 μL of PBS^-/-^ containing 10 mM D-glucose was prepared in a 2 mL cuvette (Biomart). An 880 μL volume of PiCM-G buffer (138 mM NaCl, 2.7 mM KCl, 0.6 mM CaCl_2_, 1 mM MgCl_2_, 5 mM glucose, 10 mM NaH_2_PO_4_/Na_2_HPO_4_) and 10 μL of equine ferricytochrome c (Sigma-Aldrich) was added to the suspension. The cuvettes were continuously and gently shaken during incubation at 37 °C for 10 min. PMN stimulation was achieved through the addition of either 10 μL of phorbol myristate acetate (PMA, Sigma-Aldrich) or 10 μL N-formyl-methionyl-leucyl-phenylalanine (fMLP, Sigma-Aldrich) to achieve final concentrations of 1 μM for each reagent for 30 min. The absorbance of reduced cytochrome c was recorded with the Ultrospec 3000 UV/Visible Spectrophotometer (Pharmacia Biotech) at 550 nm for a duration of 10 min.

### PMN chemotaxis

A suspension of 1 × 10^6^ PMNs in 100 μL HBSS^-/-^ containing 1% gelatin (Sigma-Aldrich) was placed on a 5% bovine serum albumin-coated 22 × 40 mm glass coverslip (Fisher Scientific) and incubated for 10 min at 37 °C. Coverslips were then inverted onto a Zigmond chamber where 100 μL of HBSS^-/-^ and 100 μL fMLP in HBSS^-/-^ containing fMLP were previously added to the right and left wells, respectively. PMN migration across the chamber was recorded with time-lapse video microscopy at 20 s intervals for a period of 15 min using an Eclipse E1000 microscope (Nikon). Captured images were analysed using cell-tracking software (Retrac, v2.1.01).

### Statistical analysis

Data were analysed by paired two-way *t*-tests using Prism (GraphPad Software, v7.00). Data is expressed as the mean and standard deviation (SD). Statistical significance was defined as *p* ≤ 0.05.
